# Synchronous Ipsilateral Occurrence of Advanced Renal Pelvic Urothelial Carcinoma With Lung Metastasis and Localized Renal Cell Carcinoma: A Case Report

**DOI:** 10.1002/ccr3.70877

**Published:** 2025-09-12

**Authors:** Taisuke Okumura, Yuichiro Kato, Akihiro Kojima, Daisuke Kato, Yohei Shimizu, Takeshi Shirakawa, Takumasa Amemiya, Tsunehiro Nenohi, Yuki Matsumoto, Masakazu Nagata, Masayasu Urushibara, Mikiko Takahashi, Minato Yokoyama, Kazuhiro Ishizaka

**Affiliations:** ^1^ Department of Urology Nissan Tamagawa Hospital Tokyo Japan; ^2^ Department of Urology Teikyo University Hospital, Mizonokuchi Kawasaki City Japan; ^3^ Department of Urology Kanto Central Hospital Tokyo Japan; ^4^ Department of Urology, Showa University School of Medicine Tokyo Japan; ^5^ Department of Diagnostic Pathology Teikyo University Hospital, Mizonokuchi Kawasaki City Japan

**Keywords:** clear cell renal cell carcinoma, enfortumab vedotin, lung metastasis, renal pelvic urothelial carcinoma, synchronous ipsilateral renal malignancies, upper tract urothelial carcinoma

## Abstract

The patient was a 92‐year‐old woman who presented with a complaint of difficulty in urination. Her abdominal computed tomography (CT) revealed two distinct types of tumors in the lower and upper poles of the right kidney, and chest CT revealed multiple lung metastatic tumors. A laparoscopic right radical nephrectomy was performed, and the lower pole tumor was diagnosed as clear cell renal cell carcinoma (RCC), whereas the upper pole tumor was renal pelvic urothelial carcinoma (UC). The lung tumor was diagnosed as metastatic UC via CT‐guided biopsy. In this case, histological examination of the primary tumor site resection and immunohistochemical examination of the lung metastases were important in diagnosing metastatic UC. Despite the challenges associated with synchronous RCC and upper tract UC (UTUC), appropriate treatment strategies, including surgical intervention and systemic chemotherapy, may improve prognosis and prolong survival. The findings contribute to the limited knowledge on synchronous RCC and UTUC, offering valuable insights to help clinicians enhance decision‐making and improve patient care in similar scenarios.

**Summary**

- This case report highlights the rare synchronous ipsilateral occurrence of advanced renal pelvic urothelial carcinoma with lung metastasis and localized renal cell carcinoma, emphasizing the importance of accurate diagnosis and appropriate treatment, including radical nephroureterectomy and targeted chemotherapy, to improve patient outcomes in complex renal malignancies.

## 1 Introduction

Renal cell carcinoma (RCC) constitutes approximately 3% of all cancers and accounts for around 90% of renal malignancies. Among its histopathological subtypes, clear cell RCC (CCRCC) is the most prevalent (70%–80%), followed by papillary (10%–15%) and chromophobe (4%–5%) [1]. Upper tract urothelial carcinoma (UTUC), including renal pelvis and ureteral cancers, is a relatively rare genitourinary malignancy, comprising 5% to 10% of urothelial cancers and less than 10% of renal tumors [2]. Pyelocaliceal tumors are approximately twice as common as ureteral tumors, with two‐thirds of UTUC cases presenting invasive disease at the time of diagnosis [3].

The synchronous ipsilateral occurrence of RCC and UTUC within the same kidney is an exceptionally rare phenomenon, first documented by Graves and Templeton in 1921 [4]. Hart et al. reviewed 23 such cases, reporting a metastasis rate of 24% at initial presentation [5]. Metastatic UTUC, in particular, has a dismal prognosis [6, 7]. This dual malignancy poses diagnostic and therapeutic challenges, particularly when accompanied by distant metastases, which further complicate prognosis and treatment strategies. Although RCC and UTUC are individually well documented, the coexistence of these malignancies in the same kidney remains sparsely reported. Furthermore, the management of metastatic lesions is unclear in such cases.

This report aims to underscore the clinical significance of synchronous ipsilateral RCC and UTUC with distant metastases, focusing on their diagnostic intricacies and therapeutic complexities. The objective is to highlight how accurate diagnosis and tailored treatment approaches, including nephrectomy and systemic therapy, can potentially improve patient outcomes in such rare and challenging cases. The findings contribute to the limited body of knowledge on synchronous RCC and UTUC, offering valuable insights for clinicians to enhance decision making and improve patient care in similar scenarios.

## 2 Case History/Examination

In May 2021, a 92‐year‐old Japanese woman was referred to our hospital after abdominal computed tomography (CT) revealed a right lower pole renal tumor, which was the suspected cause of difficulty in urination. She was being treated for atrial flutter but had no history of smoking or chemical exposure. She did not exhibit symptoms such as fever, fatigue, weight loss, gross hematuria, or flank pain. Physical examination revealed a height of 143 cm, a weight of 53.5 kg, a body mass index of 26.1 kg/m^2^, and Eastern Cooperative Oncology Group performance status score of 1.

## 3 Methods

### 3.1 Investigations

**FIGURE 1:**
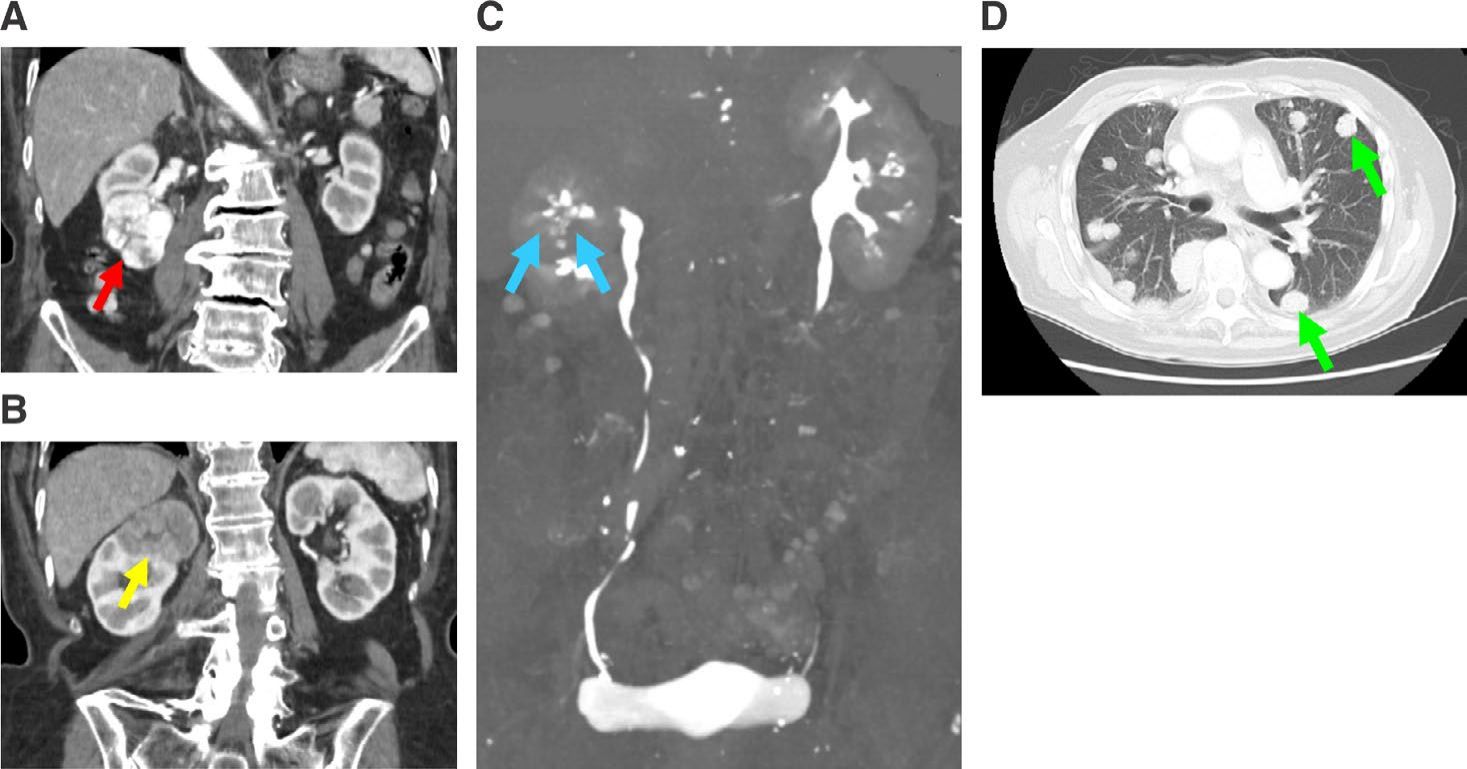
(A) Contrast‐enhanced abdominal CT reveals a strongly enhanced tumor measuring 45 × 41 × 39 mm at the lower pole of the right kidney during the arterial phase (red arrow). (B) Contrast‐enhanced abdominal CT reveals a poorly enhanced mass measuring 42 × 36 × 33 mm at the upper pole of the right kidney during the arterial phase (yellow arrow). (C) CT urography reveals a partial defect in the renal pelvis (blue arrow). (D) Plain chest CT reveals the presence of multiple lung tumors (green arrow).

Urine sediment showed 10–19 red blood cells per high‐power field, and urine cytology revealed atypical urothelial cells. Laboratory results included white blood cells at 7900/μL, neutrophils at 78.7%, hemoglobin at 10.6 g/dL, total protein at 6.8 g/dL, albumin at 3.1 g/dL, creatinine at 0.96 mg/dL, estimated glomerular filtration rate at 40.95 mL/min/1.73 m^2^, and C‐reactive protein at 21.11 mg/dL. Abdominal contrast‐enhanced CT revealed two separate tumors in the right kidney. The lower pole tumor, measuring 45 × 41 × 39 mm, exhibited strong enhancement in the arterial phase, suggesting CCRCC (Figure 1A). The upper pole mass, measuring 42 × 36 × 33 mm, showed weak contrast enhancement in the arterial phase (Figure 1B), and during the excretory phase, an irregular defect was seen in the renal calyx (Figure 1C). We suspected invasive renal pelvic urothelial carcinoma (UC), though ischemic change was also considered as a differential diagnosis. Plain chest CT revealed multiple lung tumors (Figure 1D).

### 3.2 Diagnosis and Treatment

**FIGURE 2:**
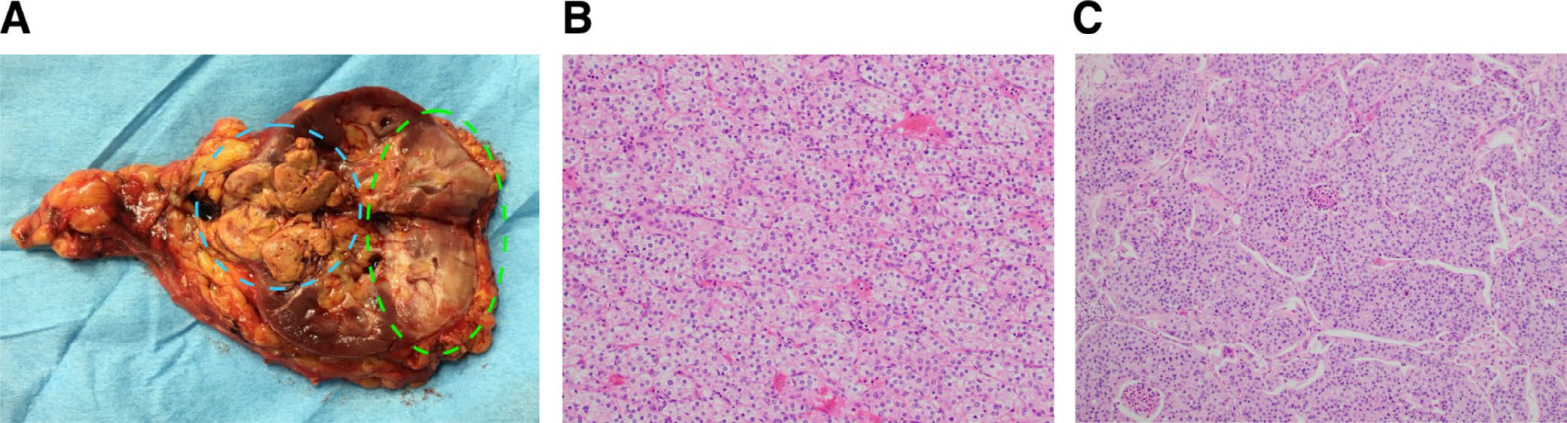
(A) Macroscopic view: cross‐section of the specimen following right radical nephrectomy shows a yellow tumor measuring 4 × 5 cm in the lower pole (dashed blue circle) and a white tumor measuring 3 × 7 cm in the upper pole (dashed green circle). (B) Microscopic view (lower pole tumor): clear cell renal cell carcinoma (WHO/ISUP grades 1–2) exhibiting an alveolar growth pattern with tumor cells having clear cytoplasm (H&E staining; magnification ×200). (C) Microscopic view (upper pole tumor): high‐grade invasive urothelial carcinoma with squamous differentiation, characterized by nests of atypical cells of varying sizes (H&E staining; magnification, ×100).

**FIGURE 3:**
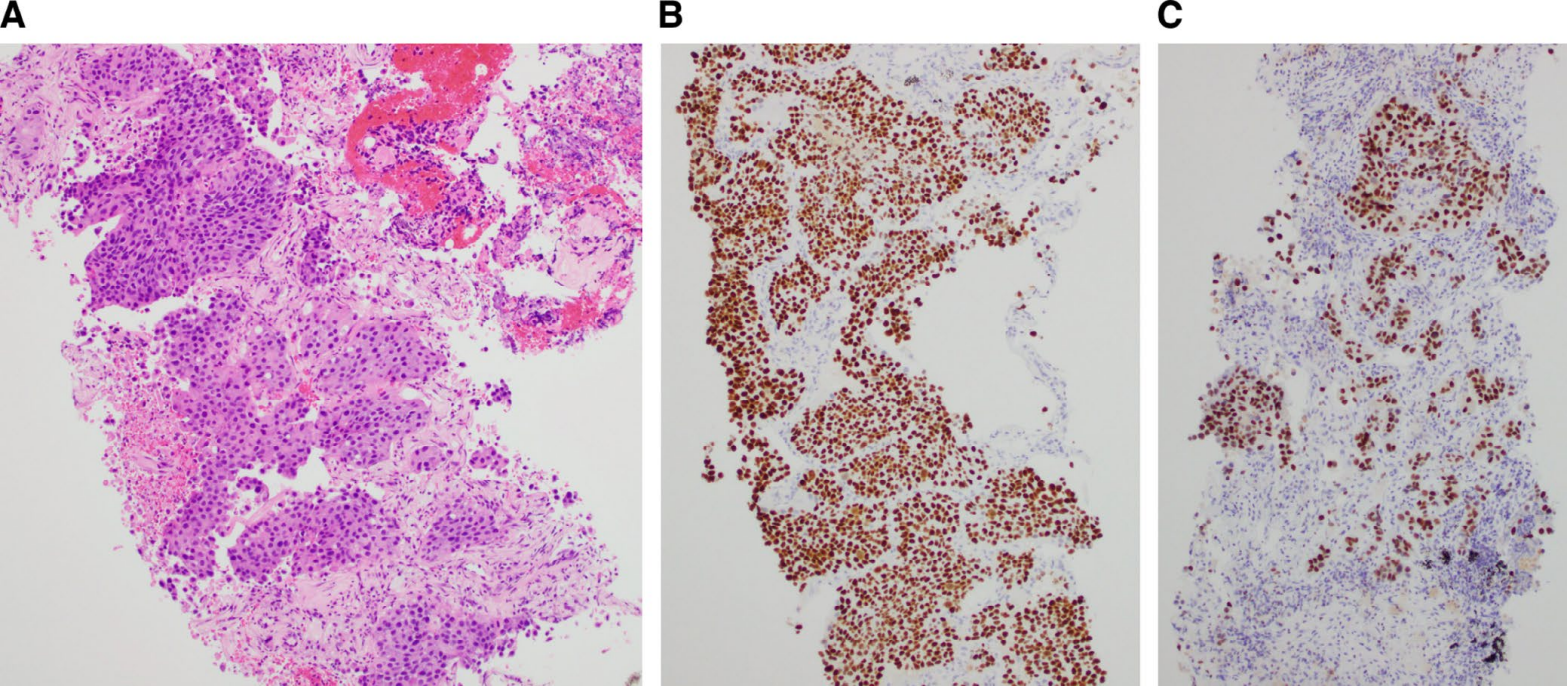
Microscopic and immunohistochemical analysis of lung tumor specimens collected by CT‐guided biopsy: (A) Nests of tumor cells with eosinophilic cytoplasm invading the pulmonary alveolar space (H&E staining; magnification, ×100). (B) Tumor cells positive for p63 (magnification, ×100). (C) Tumor cells positive for GATA3 (magnification, ×100).

The patient, diagnosed with metastatic right CCRCC or UTUC, underwent transperitoneal laparoscopic right radical nephrectomy. Due to her advanced age and physical condition, ureter and bladder cuff excision was not performed. The surgery was completed without complications, and she was discharged 12 days post‐surgery. The resected specimen showed macroscopic findings of a well‐defined yellow tumor in the lower pole and an ill‐defined white lesion in the upper pole of the right kidney (Figure 2A). Histologically, two distinct tumor types were identified. The lower pole tumor was classified as grade 2 CCRCC according to World Health Organization/International Society of Urological Pathology (WHO/ISUP) criteria, with Ly0, V1, and pT1b staging (Figure 2B). The upper pole tumor showed high‐grade invasive UC with squamous differentiation, invading the extrarenal capsule and surrounding adipose tissue, staged as pT4, Ly1, and V1 (Figure 2C). Based on the pathological findings after nephrectomy, we suspected that the UTUC had metastasized to the lungs. Furthermore, 4 weeks post‐surgery, after the patient regained sufficient strength, a CT‐guided lung tumor biopsy was performed. Microscopic examination revealed tumor cell nests with eosinophilic cytoplasm invading the pulmonary alveolar spaces. Immunohistochemical analysis was positive for p63 and GATA3, confirming the lung tumor as a metastasis of invasive UC (Figure 3A–C).

## 4 Conclusions and Results

**FIGURE 4:**
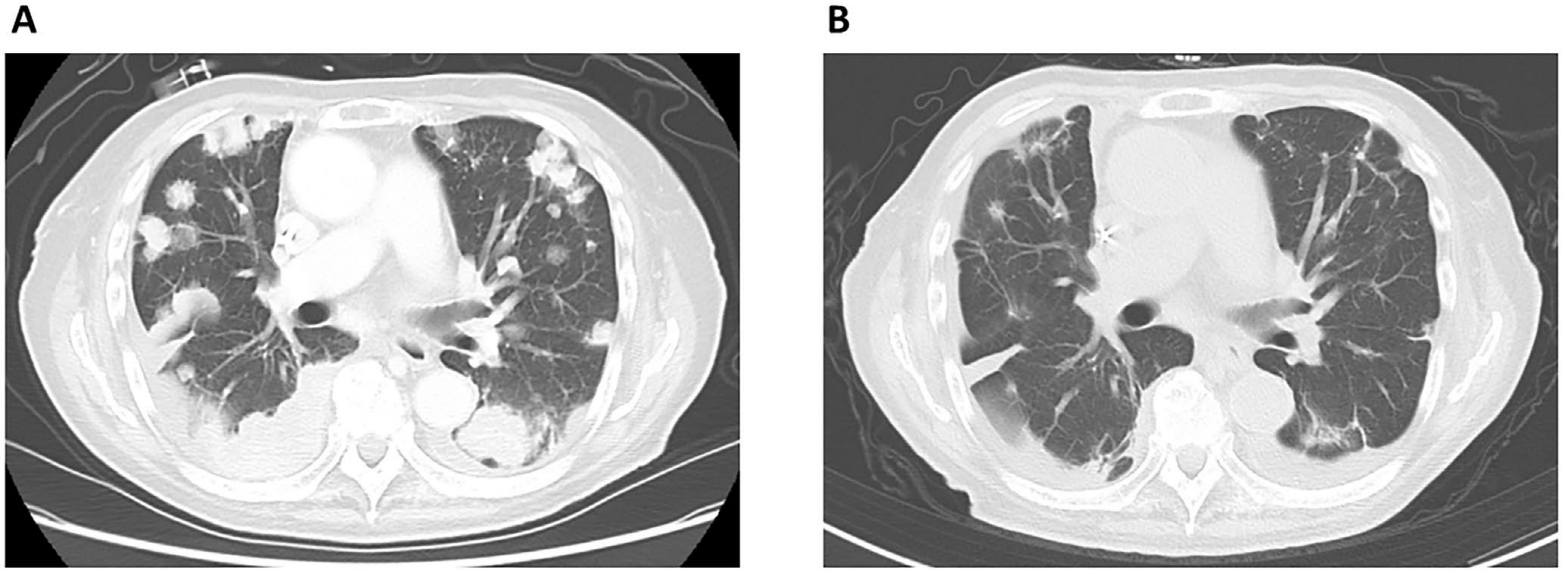
(A) After four cycles of avelumab maintenance therapy, plain chest CT shows the multiple lung tumors growth, which are more advanced than before first‐line chemotherapy. (B) After two cycles of EV therapy, plain chest CT shows significant shrinkage of the multiple lung tumors.

The patient was treated with 4 cycles of gemcitabine and carboplatin as first‐line chemotherapy because of renal function decline, followed by avelumab maintenance therapy. After 4 cycles of avelumab, treatment was changed to enfortumab vedotin (EV), an antibody‐drug conjugate targeting Nectin‐4, administered at a reduced dose (of 1.0 mg/Kg, because of the elderly) due to lung metastases growth (Figure 4A). After 2 cycles of EV therapy, lung metastatic tumors significantly shrank (Figure 4B). After 4 cycles of EV therapy, MRI revealed a new bone metastasis in the L2 spine; thus, the patient was given radiation therapy. However, after 22 cycles of EV therapy over 27 months, disease progression of spinal bone metastases led to the discontinuation of treatment, and the patient was placed on supportive care. Adverse events included grade 2 alopecia and peripheral neuropathy as well as grade 3 neutropenia. The remaining ureter and bladder cuff excision were not performed, but there was no recurrence of UC in the remaining ureter.

This case report highlights the rare occurrence of synchronous RCC and UTUC within the same kidney and illustrates the diagnostic and treatment challenges posed by these conditions. The patient's case emphasizes the importance of comprehensive imaging, accurate histological diagnosis, and the use of immunohistochemical markers in confirming metastatic UC. Despite the challenges associated with synchronous RCC and UTUC, appropriate treatment strategies, including surgical treatment, systemic chemotherapy, and targeted therapies, such as EV, may improve prognosis and prolong survival. Given the rarity of such cases, further research is needed to refine diagnostic approaches and treatment guidelines. This case contributes to the growing body of literature on synchronous cancers and underscores the importance of a multidisciplinary approach to patient management.

## 5 Discussion

Approximately 10% of patients with UTUC present with locally advanced or metastatic disease at the time of diagnosis. Metastatic UTUC has a poor prognosis, with the overall survival rate at 3 years often being less than 10% [7]. Among patients with metastatic UTUC, the lungs are the most common site of metastasis, with renal pelvic carcinoma being particularly prone to lung metastasis [8]. The standard first‐line treatment for metastatic UTUC involves platinum‐based combination chemotherapy [3]. Similarly, RCC is known for lung metastasis, which occurs in approximately 43.6% of patients with metastatic kidney cancer [9]. The synchronous occurrence of RCC and UTUC within the same kidney is rare, and when metastasis is involved, diagnosing these conditions can be especially challenging. The first report of synchronous ipsilateral RCC and UTUC was made by Graves and Templeton in 1921 [4], with more recent reviews, such as the one by Symeonidis et al. in 2022 [10], analyzing 56 cases. Histopathological types of RCC, ipsilateral synchronous occurring with UTUC include papillary and chromophobe in addition to clear cell [10, 11, 12]. A large study by Wu et al. in 2022, based on 61,564 RCC patients from the Surveillance, Epidemiology, and End Results database, found that 704 (1.1%) patients had synchronous RCC and UC. Of these, 138 (19.6%) had synchronous RCC and UTUC, and among them, 17.9% had ipsilateral RCC and UTUC. Notably, patients with papillary RCC and smaller tumors were more likely to have synchronous RCC and UTUC. Patients with synchronous RCC and UTUC had a poorer prognosis than patients with RCC only [13]. This case contributes to the existing literature by documenting the rare synchronous occurrence of RCC and UTUC in a 92‐year‐old patient. This is the oldest known patient in the literature with synchronous RCC and UTUC in the same kidney. The patient's age, coupled with the complexities of diagnosis and treatment, highlights both the challenges and significance of recognizing this rare phenomenon.

This case underscores the importance of considering the possibility of synchronous RCC and UTUC in patients with renal masses, especially when unusual imaging findings or multiple tumors are present in the same kidney. The clinical challenge lies in distinguishing between RCC and UTUC, as both can exhibit similar imaging characteristics. In our case, preoperative imaging was complicated by the presence of two distinct masses within the same kidney. CT urography remains the gold standard for diagnosing UTUC, offering the best diagnostic performance for detecting tumors in the renal collecting system [14]. However, imaging can also mimic UTUC in benign conditions, such as renal papillary necrosis or inflammatory thickening of the urinary tract wall, leading to potential misdiagnosis [15]. The use of contrast‐enhanced CT provided crucial insights into tumor localization; however, it was difficult to definitively distinguish between RCC and UTUC because of their overlapping imaging features.

The curative surgical treatment for localized RCC is partial or radical nephrectomy (RN) [1]. In previous reports, patients who underwent RN as a preoperative diagnosis of RCC had UC of the renal pelvis confirmed by intraoperative frozen section, and additional ureteral resection was supported [10, 16]. The gold standard treatment for locally advanced UTUC is radical nephroureterectomy (RNU) with bladder cuff excision [3], making it the best option for synchronous ipsilateral RCC and UTUC. A systematic review found that the diagnostic rate of RCC for percutaneous renal tumor biopsy is 92% [17], whereas a few studies reported the diagnostic rate for percutaneous biopsy in UTUC as 85%–95% [18, 19]. A preoperative biopsy can aid in the diagnosis, even with two distinct tumors in the same kidney. In our case, the diagnosis may have been made by tumor biopsy alone. However, RN enabled an accurate diagnosis of two distinct tumors and curative treatment of RCC. If systemic chemotherapy for UTUC is administered while RCC remains, the disease state may become more complicated if RCC progresses. Although the level of evidence is weak, the combination of chemotherapy with RNU offers a survival benefit compared with chemotherapy in patients with metastatic UTUC [6, 7]. In cases such as ours, resection of the primary tumor site may provide an accurate diagnosis, simplify disease management, and offer a survival benefit.

The patient in our case presented with lung metastasis, which is not always observed in other synchronous cases [10, 13]. For synchronous RCC and UTUC, histopathological confirmation is crucial. p63 and GATA3, a sensitive immunohistochemical marker for UC, were key in diagnosing lung metastasis as originating from UC in our case [20, 21, 22]. Unlike RCC, which does not express p63 and GATA3, their presence in the metastatic lung tumor confirmed that the metastasis was due to UC, not RCC. This highlights the role of immunohistochemical markers in differentiating between these two cancers and guiding treatment decisions.

This case highlights the need for further research into the optimal management strategies for synchronous RCC and UTUC. Larger, multicenter studies are essential to determine the true incidence of these rare synchronous cancers and to refine diagnostic techniques. Future research should focus on understanding the molecular and genetic underpinnings of synchronous RCC and UTUC, as well as the role of systemic therapies, such as targeted therapies and immunotherapy, in improving patient outcomes. Studies exploring the utility of early diagnosis and the impact of surgical interventions on long‐term survival would be particularly valuable. Additionally, there is a need for better guidelines on the management of patients with synchronous RCC and UTUC, especially in elderly patients, where treatment decisions may be more complex due to age‐related comorbidities and frailty.

## Author Contributions

**Taisuke Okumura:** conceptualization, writing – original draft, writing – review and editing. **Yuichiro Kato:** methodology, visualization. **Akihiro Kojima:** data curation, investigation. **Daisuke Kato:** data curation, investigation. **Yohei Shimizu:** methodology, visualization. **Takeshi Shirakawa:** investigation, resources. **Takumasa Amemiya:** data curation, investigation. **Tsunehiro Nenohi:** methodology, writing – review and editing. **Yuki Matsumoto:** data curation, investigation. **Masakazu Nagata:** resources, writing – review and editing. **Masayasu Urushibara:** conceptualization, supervision. **Mikiko Takahashi:** resources, writing – review and editing. **Minato Yokoyama:** supervision, writing – review and editing. **Kazuhiro Ishizaka:** project administration, supervision, writing – review and editing.

## Ethics Statement

As this manuscript is a case report, it does not require ethics committee approval. The case contains no patient‐identifying information and does not involve a new study necessitating International Review Board approval.

## Consent

Written informed consent was obtained from the patient for the publication of this case report and any related images.

## Conflicts of Interest

The authors declare no conflicts of interest.

## Data Availability

All data utilized or produced in this study are included within this article. Additional information can be obtained from the corresponding author upon request.
